# Estimated pneumococcal disease burden in children due to serotypes covered by different pneumococcal conjugate vaccines in five Latin American countries

**DOI:** 10.3389/fped.2026.1768403

**Published:** 2026-05-21

**Authors:** Sophie Warren, Rafael Bolanos, Lucila Rey-Ares, Juan Manuel Reyes, Jose Luis Huerta, Alana Ranzi, Rodrigo Fernandes Alexandre, Maria Gabriela Abalos, Liping Huang

**Affiliations:** 1 Global HTA, Value, & Evidence, Pfizer Inc, Cambridge, MA, United States; 2 Value & Evidence, Pfizer Peru, San Isidro, Peru; 3 Value & Evidence, Pfizer Argentina, Buenos Aires, Argentina; 4 Value & Evidence, Pfizer Colombia, Bogota, Colombia; 5 Value & Evidence, Pfizer Mexico, Ciudad de Mexico, CDMX, Mexico; 6 Value & Evidence, Pfizer Brazil, Sao Paulo, Brazil; 7 Vaccines Medical Affairs, Pfizer Argentina, Buenos Aires, Argentina; 8 Global HTA, Value, & Evidence, Pfizer Inc, Collegeville, PA, United States

**Keywords:** pneumococcal disease, disease burden, invasive pneumococcal disease, Latin America, otitis media, pneumococcal conjugate vaccines, pneumonia

## Abstract

**Introduction:**

The widespread implementation of pneumococcal conjugate vaccines (PCVs) into pediatric national immunization programs (NIPs) across Latin American countries has substantially reduced pneumococcal disease burden. While the 10-valent (PCV10) and 13-valent (PCV13) vaccines have reduced morbidity and mortality, disease caused by serotypes not included in these vaccines persists. The 15-valent (PCV15) and 20-valent (PCV20) vaccines are now available in the region. This study aimed to estimate the clinical, economic, and societal burden attributable to serotypes contained in PCV10, PCV13, PCV15, and PCV20 in children aged <5 years in Argentina, Chile, Mexico, Colombia, and Brazil

**Methods:**

Using country-specific epidemiological, cost, and population inputs, we applied a static Excel-based model to estimate annual pneumococcal disease cases, deaths, direct medical costs, and indirect societal costs resulting from caregiver productivity losses. Burden was estimated for serotypes included in PCV10, PCV13, PCV15, and PCV20, and results were interpreted in the context of the vaccine included in each country's pediatric NIP as of 2019.

**Results:**

Across the five countries, PCV20 serotypes were estimated to cause over 510,000 pneumococcal disease cases and approximately 2,700 deaths annually. This corresponded to an annual economic burden exceeding USD $182 million and a societal burden exceeding USD $34 million. Non-invasive disease, particularly pneumonia and otitis media, accounted for most of the clinical, economic, and societal burden. In countries using PCV10 in their NIP, a substantial proportion of remaining disease burden was attributable to serotypes included in PCV13 but not PCV10. Across all countries, serotypes unique to PCV15 contributed little additional burden beyond PCV13, whereas serotypes unique to PCV20 accounted for a substantial share of remaining disease burden, particularly in PCV13 NIP countries.

**Conclusion:**

Despite the success of PCV10 and PCV13 in reducing pneumococcal disease in Latin America, a considerable clinical, economic, and societal burden remains due to serotypes included in higher-valent vaccines. Broader serotype coverage offered by PCV20 has the potential to address this unmet burden and further reduce the public health and economic impact of pneumococcal disease in young children across the region.

## 1 Introduction

**Figure 1:**
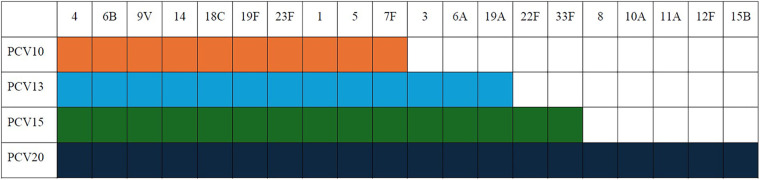
Serotype composition of PCVs available in Latin America. PCV, pneumococcal conjugate vaccine.

Pediatric pneumococcal disease continues to be a leading cause of vaccine-preventable morbidity and mortality in Latin America. Invasive pneumococcal disease (IPD) incidence among children <5 years old in the region is estimated to be around 24 per 100,000, with case fatality rates of IPD and pneumococcal pneumonia ranging from 4% to7% (1). In 2011, the Pan American Health Organization (PAHO) recommended the introduction of pneumococcal conjugate vaccines (PCVs) into the national immunization programs (NIPs) of Latin American countries. Since then, two PCVs have been included in most NIPs: a 10-valent PCV (PCV10; GlaxoSmithKline plc.) and a 13-valent PCV (PCV13; Pfizer, Inc.) (2, 3). Two other higher-valent PCVs, namely a 15-valent PCV (PCV15; Merck) and a 20-valent PCV (PCV20; Pfizer, Inc.), have recently become available in some Latin America countries and may soon become available in others. PCV10 contains 10 pneumococcal serotypes (1, 4, 5, 6B, 7F, 8, 9 V, 14, 18C, and 19F), PCV13 contains PCV10 serotypes plus serotypes 3, 6A, and 19A, PCV15 contains PCV13 serotypes plus serotypes 22F and 33F, and PCV20 contains PCV15 serotypes plus serotypes 8, 10A, 11A, 12F, and 15B (Figure 1).

Since the implementation of PCV10 and PCV13 in pediatric NIPs, there has been a marked reduction in pneumococcal disease incidence both globally and within Latin American countries, especially in settings with high vaccination coverage. In Argentina, hospitalization rates for IPD and community-acquired pneumonia in children <5 years old decreased by more than half in the two years after PCV13 was introduced (4). Similarly, Mexico experienced a 73% decrease in IPD mortality and 68% decrease in overall IPD incidence following PCV13 implementation (5). Despite offering protection against fewer serotypes than PCV13, PCV10 has also demonstrated an impact on pediatric pneumococcal disease. Chile introduced PCV10 into the NIP in 2011 and, after two years of PCV10 use, the number of pneumonia-related hospitalizations and deaths among infants were substantially reduced (6).

Despite the success of PCV10 and PCV13, a significant burden of pneumococcal disease remains, especially disease caused by serotypes not contained in PCV10 or PCV13. Higher-valent PCV formulations such as PCV15 and PCV20 have the potential to address this unmet need by providing additional protection against emerging serotypes not covered by PCV10 or PCV13. Although PCV15 and PCV20 have been implemented in NIPs across different regions since 2023, there is currently no available data on their real-world effectiveness in children. Nevertheless, both vaccines are expected to further reduce pneumococcal disease cases and deaths, as well as their associated costs.

Pneumococcal epidemiology and circulating serotypes vary by region and are driven by NIP recommendations, vaccine uptake, and the vaccine-type serotypes that were historically included in the NIP. Considering the availability of higher-valent PCVs, estimations of the burden associated with vaccine serotypes can help inform vaccine recommendations and reimbursement decisions. As such, this study aimed to estimate the annual clinical, economic, and societal burden due to *Streptococcus pneumoniae* serotypes contained in PCV10, PCV13, PCV15, and PCV20 in children <5 years old in five Latin American countries.

## 2 Methods

### 2.1 Overview

We used an Excel-based model to estimate the annual clinical, economic, and societal burden of pneumococcal disease in children <5 years old in Argentina, Chile, Mexico, Colombia, and Brazil. The selection of these five countries was based on the availability of epidemiologic data from observational studies and comprehensive serotype coverage information obtained through large-scale, population-based IPD surveillance to inform the model, as well as the intent to ensure representation across diverse regions of Latin America. The model defines clinical, economic, and societal burden as follows:

- *Clinical burden*: estimated annual number of cases and deaths attributable to the specific pneumococcal serotypes included in each PCV. This represents the disease incidence and mortality that could potentially be prevented through vaccination in children under 5 years old.
- *Economic burden*: estimated annual direct medical costs associated with treating pneumococcal disease cases caused by vaccine-type serotypes, expressed in 2023 USD. This includes expenses related to hospitalization, medical treatment, and healthcare resources required for managing these cases.
- *Societal burden*: estimated indirect costs resulting from productivity loss, measured as caregivers’ missed hours of paid work while caring for children with pneumococcal disease. This reflects the broader impact of disease on families and society, beyond direct medical expenses.

The model does not consider vaccination uptake or vaccine effectiveness and intends to only estimate the annual cases and costs that could be preventable by serotypes included in each vaccine. We categorized countries based on the vaccine included in the pediatric NIP as of December 31st, 2019. Therefore, Mexico, Chile, and Argentina were categorized as PCV13 NIP countries and Brazil and Colombia as PCV10 NIP countries.

### 2.2 The model

The same model has been used in previous assessments of pneumococcal disease burden in other countries, including the United States and multiple countries across Europe and Asia (7–10). This model was informed by previously conducted studies and does not contain any new studies with human participants or animals performed by any of the authors. All model inputs were based on a point of estimate and therefore the results of the analysis do not present confidence intervals.

**Figure 2:**
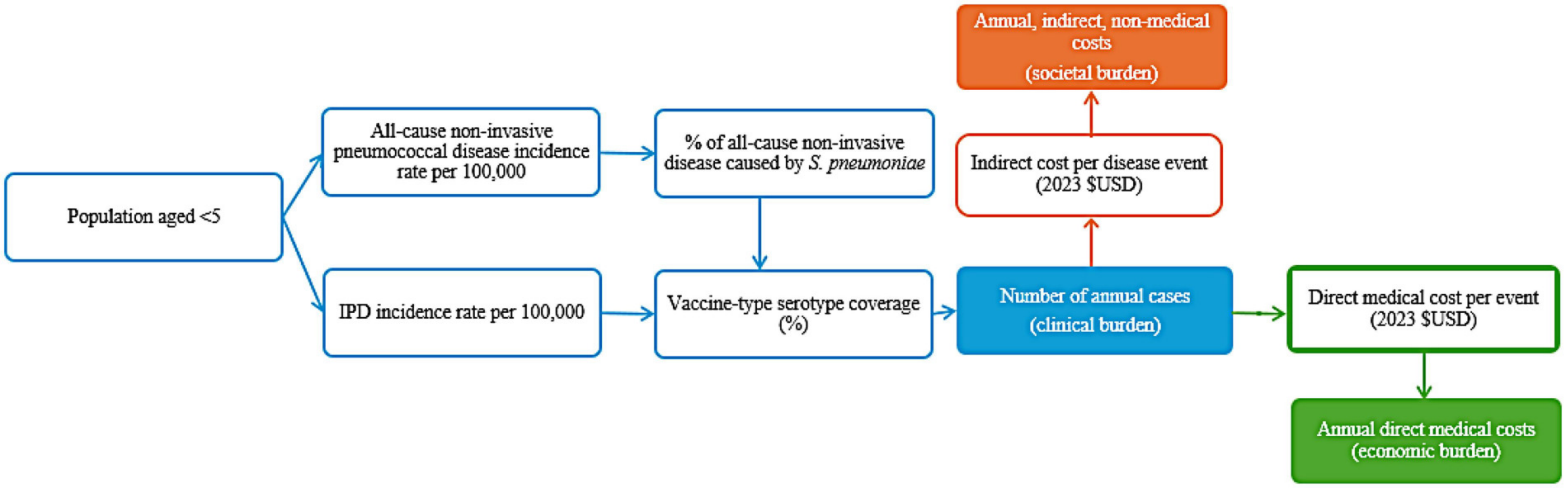
Visual overview of model structure depicting how inputs were combined to calculate annual clinical, economic, and societal burden. Epidemiologic inputs, denoted in the boxes outlined in blue, are multiplied to estimate the clinical burden. The clinical burden is then multiplied by the direct and indirect cost per event to determine the economic and societal burden, respectively. Additional detail on model calculations can be found in the Supplementary Materials. IPD, invasive pneumococcal disease; USD, United States dollar.

Briefly, the model used epidemiological, economic, and population data to aggregate the total number of IPD, pneumonia, and OM cases in each country, stratified by local serotype coverage for each PCV. The number of disease cases is then used to calculate the economic and societal burden (Figure 2). We employed a human capital approach to estimate hours of lost caregiver productivity per episode of pediatric pneumococcal disease and consequent societal burden. A detailed explanation of all model calculations can be found in the Supplementary Materials.

To estimate the annual burden of pneumococcal disease in children <5 years old for each country, the model combined the number of disease cases, deaths, direct medical costs, and indirect societal costs for each disease type. These totals were calculated for serotypes covered by the PCV10, PCV13, PCV15, and PCV20 vaccines.

### 2.3 Model inputs and assumptions

The model used country-specific epidemiology and cost data to estimate clinical, societal, and economic burden. Key model assumptions are listed in Table 1. Input values and sources for each country are presented in Table 2 and described in further detail throughout the methods.

**Table 1:** Overview of key assumptions for the burden of disease model.

Domain	Assumption
Study perspective	Societal perspective including clinical outcomes, direct costs, and indirect costs
Time horizon	One-year
Population	Children aged <5 years
Non-invasive disease serotype distribution	Assumed identical to IPD serotype distribution
Pneumococcal attribution	29.1% of all-cause pneumonia and OM attributable to *S. pneumoniae*
Epidemiologic inputs	Incidence rates and case fatality rates assumed constant within each country
Age extrapolation	Age-specific inputs extrapolated to <5 years when required to avoid data manipulation

IPD, invasive pneumococcal disease; OM, otitis media.

**Table 2:** Population, epidemiology, and cost inputs used to estimate the burden of disease in children <5 years of age.

	Argentina	Mexico	Chile	Colombia	Brazil
Population <5	3,668,251 (37)	10,047,365 (38)	1,166,146 (39)	4,373,565 (40)	12,704,860 (41)
Incidence per 100,000					
IPD	10.0 (22, 42)	18.6 (22)	13.3 (29)	12 (30, 31)^b^	21.0 (34)
Inpatient PNE	329.7 (23)	801.5 (28)	340.0 (29)	1,312.8 (30, 31)^c^	3,433.0 (35)
Outpatient PNE	123.2 (23)	6,412.0 (27)	15,227.8 (24)	5,133.3 (30, 31)^c^	3,643.1 (24)
OM	8,493.0 (24)	855.3 (27)	16,260.0 (24)	2,574.3 (30, 31)^d^	761.2 (36)
% IPD presenting as meningitis	23 (42)	9 (14)	17 (29)	11 (11)	45 (11)
% non-invasive disease attributable to *S. pneumoniae* (20)	29.1	29.1	29.1	29.1	29.1
CFRs^a^ (%)					
Meningitis	14.3 (24)	14.7 (27)	4.0 (29)	26.0 (33)	13.0 (34)
Bacteremia	1.5 (25)	4.5 (27)	4.0 (29)	14.0 (33)	13.0 (34)
PNE	3.5 (24)	3.0 (27)	0.5 (24)	9.0 (33)	1.0 (34)
Direct medical cost per disease (2023 $USD)					
Bacteremia	3,530 (21)	5,818 (43)	608 (24)	5,160 (44)	567 (34)
Meningitis	2,741 (21)	10,853 (43)	2,427 (24)	7,305 (44)	699 (34)
Inpatient PNE	2,158 (21)	4,245 (43)	706 (24)	1,275 (44)	237 (34)
Outpatient PNE	150 (21)	336 (43)	65 (24)	43 (44)	76 (34)
OM	31 (21)	2,602 (43)	28 (24)	25 (44)	76 (34)
Indirect cost per disease (2023 $USD)					
IPD	283	228	367	161	240
Inpatient PNE	291	180	306	147	160
Outpatient PNE	100	60	153	43	80
OM	20	12	31	9	16

OM, otitis media; CFR, case fatality rate; IPD, invasive pneumococcal disease; PNE, pneumonia; USD, United States dollar.

a CFR for meningitis and bacteremia assumed to be the same if data only presented CFR for overall IPD.

b ICD-10 codes: A39.0, A39.4, A39.9, A40.3, A41.3, A41.5, A41.8, A41.9, A49.2, A49.8, A49.9, B95.3, G00.0, G00.1, G00.2, G03.9.

c ICD-10 codes: J13, J14, J15.6, J15.8, J15.9, J16.8, J18.0, J18.1, J18.8, J18.9, J22.

d ICD-10 codes: H65.0-H65.4, H65.9-H66.4 L, H66.9.

#### 2.3.1 Epidemiology

The epidemiological inputs used in the model included disease incidence rates per 100,000 for IPD (defined as meningitis and bacteremia), all-cause pneumonia, and all-cause OM, case-fatality rates (CFRs), and vaccine-type serotype coverage, compiled from published literature or regional surveillance reports (Table 2). To avoid data manipulation and to increase transparency, in some instances incidence rates reported for specific age stratifications in children, e.g., < 1- or 0–2-year-olds, were assumed to be the same as that for <5-year-olds.

##### 2.3.1.1 Serotype coverage

IPD serotype coverage estimates were informed by published literature or country-specific Sistema Regional de Vacunas (SIREVA) data from 2019 (11). In Argentina, the total number of isolates from 2017 to 2019 was used due to low case counts in 2019 (11). Pre-2020 data were prioritized to minimize the impact of COVID-19-related social distancing on serotype coverage, as global IPD case numbers declined between 2020 and 2022 (12–14).

The serotype coverage for non-invasive pneumococcal disease was assumed to be the same as that for IPD. Serotype data for non-invasive pneumococcal disease are limited and rarely reported. This is especially true for Latin America but is a measurement challenge for pneumococcal disease globally. For example, administrative claims databases tend to underreport the proportion of all-cause pneumonia caused by pneumococcus because there are no highly accurate tools for confirming a diagnosis for pneumococcal pneumonia (15). Additionally, serotyping for OM typically only occurs in more severe cases, such as a spontaneous rupture, or is otherwise collected in a highly invasive manner, such as an intentional puncture of the tympanic membrane. Due to these limitations, we employed this assumption that is frequently used in pneumococcal disease burden assessments and economic evaluations of pneumococcal vaccines (10, 16–19).

The model assumed that 29.1% of all-cause pneumonia and OM cases were attributable to *S. pneumoniae*. This assumption is supported by evidence from a systematic literature review which found that *S. pneumoniae* accounted for, on average, 29.1% of OM cases in South America (20). A one-way deterministic sensitivity analysis tested this assumption using minimum and maximum estimates in South American countries from the same source.

##### 2.3.1.2 Disease incidence and CFR

###### 2.3.1.2.1 Argentina

Incidence rates for IPD, inpatient pneumonia, outpatient pneumonia, and OM were aligned with a recently published cost-effectiveness analysis (CEA) of PCV20 vs. PCV15 and PCV13 in Argentina (21). To inform IPD incidence, case count data from SIREVA 2019 were adjusted to account for potential underreporting using data from Nieto Guevara and Guzman-Holst, 2021 (13, 22). All-cause pneumonia incidence was obtained from Gentile et al. (23) and the proportion of outpatient and inpatient cases (27.2% and 72.8%, respectively) was used to estimate outpatient and inpatient incidence. OM incidence and CFR for meningitis were informed by Garcia Marti et al. (24), while CFRs for bacteremia and inpatient pneumonia were sourced from Urueña et al. (25).

###### 2.3.1.2.2 Mexico

Disease incidence rates for Mexico were also aligned with a recently published CEA of PCV20 vs. PCV13 and PCV15 (26), using reported estimates from the <1 age group. To derive IPD incidence rates, case count data from SIREVA were adjusted in the same manner as done for the Argentina data to account for potential underreporting. CFRs for bacteremia, meningitis, and inpatient pneumonia as well as incidence rates for outpatient pneumonia and OM were sourced from Wasserman et al. (27). The incidence rate for inpatient pneumonia was calculated using the number of all-cause inpatient pneumonia cases in Mexico from Statista and population data (28).

###### 2.3.1.2.3 Chile

IPD incidence, inpatient pneumonia incidence, and IPD CFR were retrieved from Alvarado et al. (29), an observational study which assessed the impact of PCV10 on these outcomes between 2009 and 2015. Outpatient pneumonia incidence, OM incidence, and inpatient pneumonia CFR were sourced from Garcia Marti et al. (24).

###### 2.3.1.2.4 Colombia

IPD incidence in Colombia was estimated using meningitis and bacteremia cases reported in the national Individual Health Service Delivery Records (RIPS) database for 2022, identified through ICD-10 codes, along with the population under 4 years of age reported by the National Administrative Department of Statistics (DANE) for the same year (30, 31). Estimates were adjusted using the 43.3% proportion of *S. pneumoniae* identified in meningitis cases reported by Camacho-Moreno et al. (32). Similarly, inpatient pneumonia, outpatient pneumonia, and otitis media cases were extracted from the RIPS database for 2022 using ICD-10 codes (30). CFRs for IPD and inpatient pneumonia were sourced from Camacho-Moreno et al. (33).

###### 2.3.1.2.5 Brazil

IPD incidence rate, IPD CFR, and inpatient pneumonia CFR were derived from Perdrizet et al. 2021, using the estimate for 0–2-year-olds (34). Inpatient and outpatient pneumonia incidence rates were sourced from Andrade et al. (35) and Garcia Marti et al. 2013, respectively (24). OM incidence was informed by Sartori et al. (36).

#### 2.3.2 Direct costs

Country-specific costs per disease episode were extracted from published data, inflated to 2023 values as necessary using Consumer Price Index inflation rates, then converted to USD based on national currency conversion rates.

#### 2.3.3 Indirect costs

Societal burden was calculated as indirect costs, defined as productivity loss (cost of missed hours of paid work) incurred by caregivers of infected children. The model employed a methodology described by Li et al. (8), updated to consider missed working hours rather than working days. The full methodology for calculating the indirect costs is described in the Supplementary Materials.

### 2.4 Analyses

The model estimated the annual disease cases and deaths, the disease-related direct medical cost and indirect cost based on the inputs listed in Table 2. The base-case analysis assumed that 29.1% of OM and pneumonia cases were due to S*. pneumoniae*. Alternative scenarios evaluated lower (16.0%) and higher (42.2%) attribution assumptions, based on data from South America (20).

## 3 Results

### 3.1 Overview

The results present the clinical, economic, and societal burden of PCV10, PCV13, PCV15, and PCV20 serotypes. The term “PCV10/PCV13/PCV15/PCV20 serotypes” is inclusive of all serotypes covered by each vaccine. Incremental burden is described as cases, deaths, or costs due to the additional serotypes covered by one vaccine relative to a lower-valent option, which is denoted as PCV13-10 serotypes (3, 6A, and 19A), PCV15-13 serotypes (22F and 33F), and PCV20-15 serotypes (8, 10A, 11A, 12F, 15B).

### 3.2 Serotype coverage

**Figure 3:**
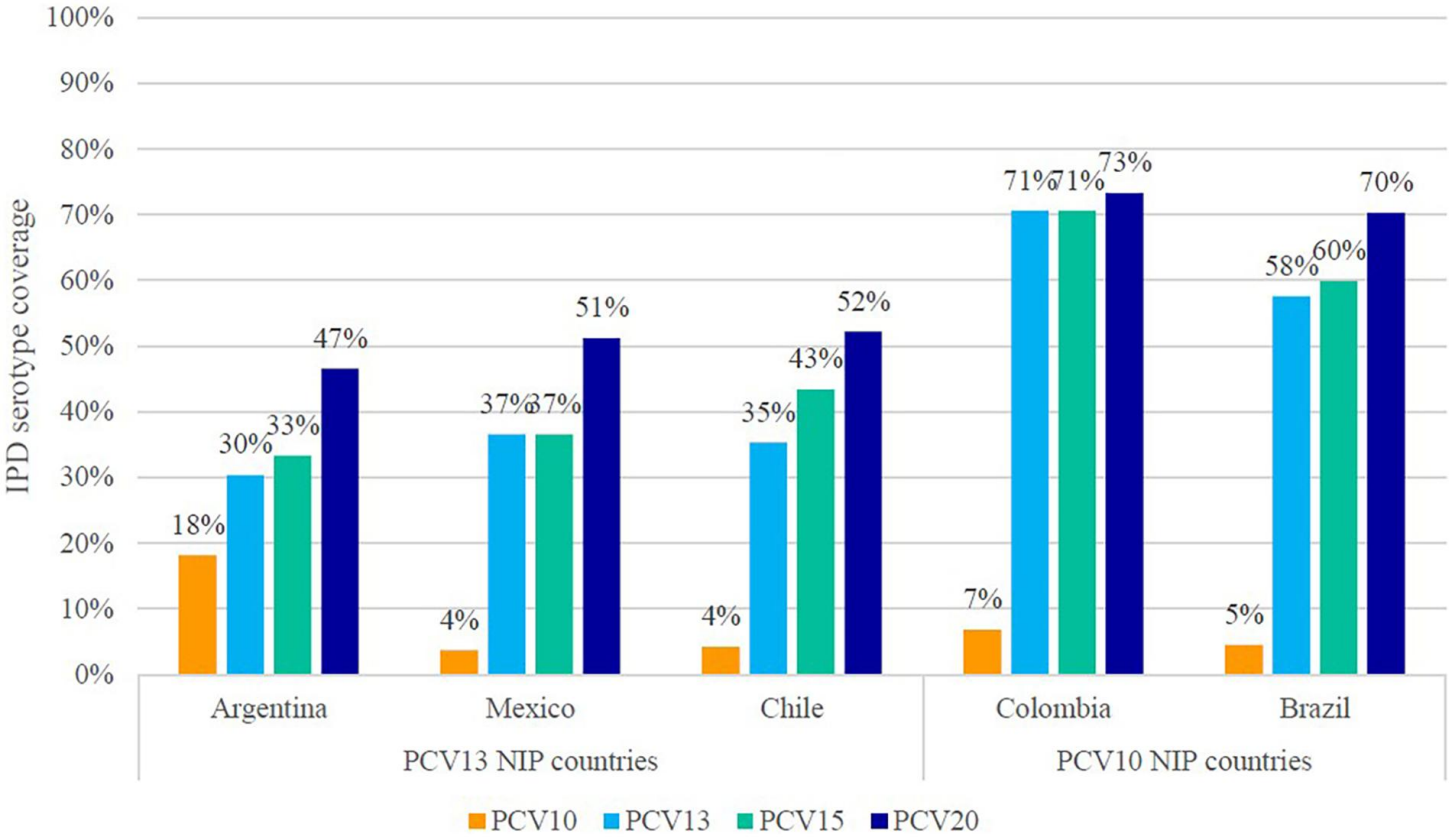
IPD serotype coverage of PCV10, PCV13, PCV15, and PCV20 in children <5 years of age, based on SIREVA data. IPD, invasive pneumococcal disease; NIP, national immunization program; PCV, pneumococcal conjugate vaccine; SIREVA, Sistema Regional de Vacunas.

Figure 3 presents the serotype coverage for each country and serotype specific estimates of disease coverage are presented in Supplementary Table 2. The PCV13-10 serotypes were responsible for a greater proportion of IPD in PCV10 NIP countries (53% for Brazil and 64% for Colombia) than PCV13 NIP countries (12% for Argentina, 31% for Chile, and 33% for Mexico). Across the five countries, compared with PCV13 serotypes, there was little to no disease caused by PCV15-13 serotypes. In Mexico and Colombia, the serotype coverage for both PCV13 and PCV15 were the same, whereas in Argentina, Brazil, and Chile, the difference of serotype coverage of PCV13 vs. PCV15 ranged from only 3% to 8%. PCV20-15 serotype coverage was slightly higher in the PCV13 NIP countries (9% to 15%) than that reported in the PCV10 NIP countries (3% to 10%). Overall, the remaining vaccine-type IPD in PCV10 NIP countries was higher than in PCV13 NIP countries, ranging from 70% to 73% vs. 47% to 52%.

### 3.3 Clinical burden

Table 3 presents the total estimated annual cases and deaths in children aged <5 years for each pneumococcal disease manifestation attributable to serotypes covered by each PCV. Across all five countries, the model estimated PCV20 serotypes were responsible for a total of 3,470 IPD cases, 381,227 pneumonia cases, and 127,663 OM cases annually. Additionally, PCV20 serotypes were estimated to cause an annual 2,776 deaths.

**Table 3:** Estimated annual clinical burden, including pneumococcal disease cases and deaths, attributable to the serotypes included in PCV10, PCV13, PCV15, and PCV20.

	PCV13 NIP Countries			PCV10 NIP Countries	
	Argentina	Mexico	Chile	Colombia	Brazil
Population aged 0–4	3,668,251	10,047,365	1,166,146	4,373,565	12,704,860
IPD cases					
PCV10 serotypes	66	68	7	36	120
PCV13 serotypes	112	682	55	371	1,537
PCV15 serotypes	122	682	67	371	1,598
PCV20 serotypes	171	957	81	385	1,876
Inpatient PNE cases					
PCV10 serotypes	637	855	48	1,136	5,692
PCV13 serotypes	1,072	8,553	407	11,796	72,856
PCV15 serotypes	1,172	8,553	501	11,796	75,765
PCV20 serotypes	1,640	11,998	602	12,247	88,919
Outpatient PNE cases					
PCV10 serotypes	238	6,843	2,170	4,443	6,040
PCV13 serotypes	401	68,428	18,241	46,124	77,312
PCV15 serotypes	438	68,428	22,427	46,124	80,400
PCV20 serotypes	613	95,986	26,975	47,888	94,359
OM cases					
PCV10 serotypes	16,400	913	2,317	2,229	1,266
PCV13 serotypes	27,624	9,128	19,478	23,140	16,210
PCV15 serotypes	30,190	9,128	23,947	23,140	16,857
PCV20 serotypes	42,247	12,804	28,803	24,025	19,784
Total cases					
PCV10 serotypes	17,341	8,679	4,543	7,843	13,118
PCV13 serotypes	29,209	86,791	38,181	81,431	167,915
PCV15 serotypes	31,922	86,791	46,942	81,431	174,620
PCV20 serotypes	44,671	121,745	56,461	84,545	204,938
Deaths					
PCV10 serotypes	25	29	1	108	73
PCV13 serotypes	42	293	4	1,119	928
PCV15 serotypes	46	293	5	1,119	965
PCV20 serotypes	65	411	6	1,161	1,133

OM, otitis media; IPD, invasive pneumococcal disease; NIP, national immunization program; PCV, pneumococcal conjugate vaccine; PNE, pneumonia.

In Colombia and Brazil, more than 70,000 and 150,000 cases, respectively, could be attributed to the PCV13-10 serotypes, meaning PCV13 could theoretically prevent 9–12 times more cases in those countries. In Argentina, Mexico, and Chile, PCV13 serotypes were estimated to cause 0.7–9 times more cases, respectively, than PCV10 serotypes. There was relatively little or no difference between case numbers attributable to PCV13 and PCV15 serotypes across the five countries, with the PCV15 serotypes estimated to cause about 2,700, 8,700, and 380 more cases than PCV13 serotypes in Argentina, Chile, and Brazil, respectively. In Mexico and Colombia, there were no additional cases estimated due to the PCV15-13 serotypes. The PCV20-15 serotypes contributed to a high portion of the total clinical burden, including over 12,000 cases in Argentina, 34,000 in Mexico, 9,500 in Chile, 3,000 in Colombia, and 30,000 in Brazil.

Incremental annual deaths presented similar patterns to the differences in cases. In the PCV10 NIP countries, the greatest annual number of deaths were estimated to be due to the PCV13-10 serotypes. In Colombia and Brazil, this resulted in 1,011 and 855 additional disease-related deaths annually, whereas the difference ranged from 3 to 264 annual deaths in PCV13 NIP countries. No incremental annual deaths due to the PCV15-13 serotypes were estimated in Mexico and Colombia. In Argentina, Brazil, and Chile, PCV15-13 serotypes were estimated to only cause 1–4 additional deaths. The PCV20-15 serotypes were estimated to cause an additional 1 to 168 deaths annually across the five countries (Table 3).

**Figure 4:**
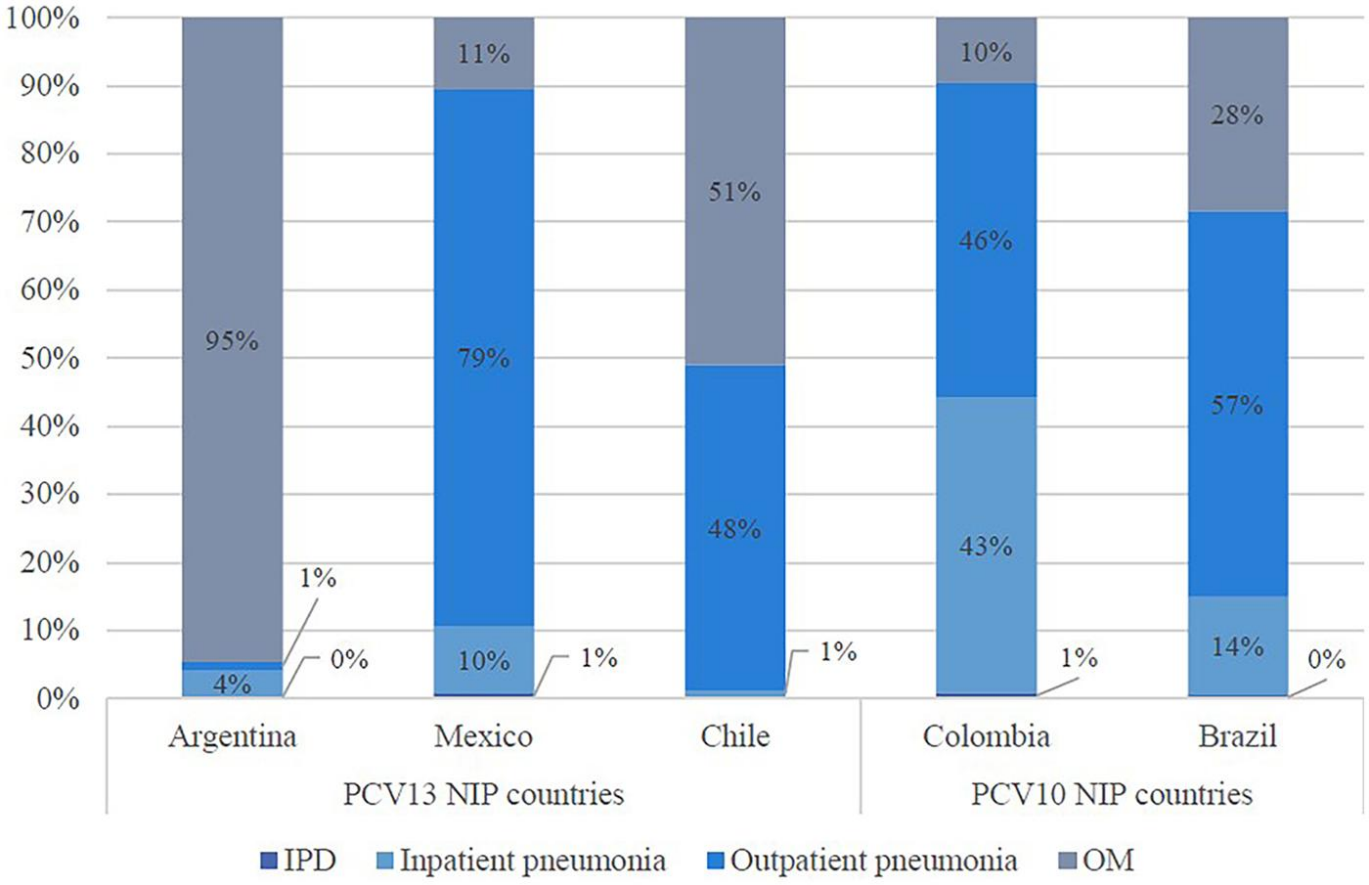
Estimated proportion of annual total pneumococcal disease cases by disease state. OM, otitis media; IPD, invasive pneumococcal disease; NIP, national immunization program; PCV, pneumococcal conjugate vaccine.

Non-invasive disease contributed to the greatest clinical burden across all PCV13 NIP countries (Figure 4). The majority of the annual pneumococcal disease burden in Argentina and Chile was estimated to be OM, representing 95%, and 51% of all disease cases, respectively. Most of the annual disease burden in Mexico and Colombia were estimated to be due to pneumonia, specifically outpatient pneumonia (79% and 46%, respectively). In Brazil, inpatient pneumonia accounted for the greatest proportion of clinical burden (43%).

### 3.4 Economic burden

Across all countries and disease manifestations, there was an incremental increase in economic burden associated with serotypes covered by higher-valent PCVs. PCV10 serotypes were estimated to be associated with annual direct medical costs ranging from $0.25 to $8.7 million (Table 4). In PCV10 NIP countries, the PCV13-10 serotypes were associated with $17.7 to $23.3 million and in the PCV13 NIP countries, they were associated with an additional $1.5 to $78.6 million. The difference in annual direct medical cost caused by PCV13 serotypes vs. PCV15 serotypes was relatively small, at $0.3 million in Argentina, $0.2 million in Brazil, and $0.5 million in Chile. In Mexico and Colombia, there were no difference in costs. Compared with the direct medical cost associated with PCV15-13 serotypes, the difference attributable to the PCV20-15 serotypes was greater: in Argentina it was $1.6 million, in Brazil $4.6 million, in Chile $0.5 million, in Colombia $0.75 million, and in Mexico $35.2 million.

**Table 4:** Estimated annual economic and societal burden due to pneumococcal disease cases attributable to the serotypes included in PCV10, PCV13, PCV15, and PCV20 (in million USD).

	PCV13 NIP Countries			PCV10 NIP Countries	
	Argentina	Mexico	Chile	Colombia	Brazil
Economic burden^a^					
PCV10 serotypes	$2.13	$8.73	$0.25	$1.89	$1.98
PCV13 serotypes	$3.59	$87.28	$2.07	$19.60	$25.33
PCV15 serotypes	$3.93	$87.28	$2.55	$19.60	$26.34
PCV20 serotypes	$5.50	$122.44	$3.06	$20.35	$30.91
Societal burden^b^					
PCV10 serotypes	$0.56	$0.59	$0.42	$0.39	$1.01
PCV13 serotypes	$0.94	$5.91	$3.53	$4.00	$12.99
PCV15 serotypes	$1.02	$5.91	$4.34	$4.00	$13.51
PCV20 serotypes	$1.43	$8.29	$5.22	$4.15	$15.85

NIP, national immunization program; PCV, pneumococcal conjugate vaccine.

a Economic burden inclusive of direct medical costs due to each pneumococcal disease state, including IPD, inpatient pneumonia, outpatient pneumonia, and OM.

b Societal burden inclusive of indirect costs due productivity loss resulting from each pneumococcal disease state, including IPD, inpatient pneumonia, outpatient pneumonia, and OM.

Economic burden by disease state in each country is reported in Supplementary Table 3. In Argentina, Mexico, Colombia, and Brazil, the greatest proportion of economic burden resulted from inpatient pneumonia, with total estimated annual direct medical costs due to PCV20 serotypes amounting to $3.6 million, $50.1 million, $15.6 million, and $21.0 million, respectively. In Chile, outpatient pneumonia was the largest cause of economic burden, with annual direct medical costs of $1.8 million due to PCV20 serotypes.

### 3.5 Societal burden

Hours of productivity loss per case are shown in Supplementary Table 1. IPD resulted in more hours of productivity loss and the greatest indirect cost per case in all countries. However, non-invasive disease resulted in higher societal burden due to the high case numbers.

PCV10 serotypes were estimated to be associated with annual societal burden of $0.6 million in Argentina, $0.6 million in Mexico, $0.4 million in Chile, $1.0 million in Brazil, and $0.4 million in Colombia (Table 4). In PCV10 NIP countries, PCV13-10 serotypes contributed to $3.6 to $12 million in additional societal burden, while in PCV13 NIP countries they accounted for an additional $0.4 to $5.3 million. The incremental societal burden caused by the PCV15-13 serotypes was small, at $0.1 million in Argentina, $0.5 million in Brazil, and $0.8 million in Chile. In Mexico and Colombia, no difference was observed. The societal burden associated with the PCV20-15 serotypes was $0.4 million in Argentina, $2.4 million in Mexico, $0.9 million in Chile, $2.34 million in Brazil, and $0.15 million in Colombia.

Societal burden by disease state in each country is reported in Supplementary Table 4. In Mexico, Chile, and Colombia outpatient pneumonia resulted in the greatest estimated societal burden, at $5.8 million, $4.1 million, and $2.08 million, respectively. Inpatient pneumonia constituted the greatest societal burden in Brazil ($14.2 million). In Argentina, the greatest societal burden came from OM, at $1.4 million.

### 3.6 Scenario analysis

Across all countries, absolute changes in total cases, direct medical costs and indirect costs, which inferred economic burden and societal burden, were directionally consistent with the attribution assumptions applied in the scenario analysis (Table 5). When the proportion of pneumonia and OM caused by *S. pneumoniae* was decreased to 16.0% or increased to 42.2%, the total clinical, economic, and societal burden changed by approximately 40% to 45% in either direction.

**Table 5:** Estimated annual clinical, economic, and societal burden due to pneumococcal disease attributable to the serotypes included in PCV10, PCV13, PCV15, and PCV20 when varying the proportion of non-invasive disease attributable to *S. pneumoniae*.

	PCV13 NIP Countries			PCV10 NIP Countries	
	Argentina	Mexico	Chile	Colombia	Brazil
Lower assumed proportion of *S. pneumoniae*					
Clinical burden^a^					
PCV10 serotypes	9,565	4,803	2,501	4,328	7,289
PCV13 serotypes	16,110	48,027	21,018	44,940	93,301
PCV15 serotypes	17,606	48,027	25,841	44,940	97,027
PCV20 serotypes	24,638	67,370	31,080	46,658	113,873
Economic burden^b^ (in million USD)					
PCV10 serotypes	$1.27	$4.99	$0.14	$1.13	$1.13
PCV13 serotypes	$2.15	$49.90	$1.16	$11.68	$14.40
PCV15 serotypes	$2.34	$49.90	$1.42	$11.68	$14.98
PCV20 serotypes	$3.28	$70.00	$1.71	$12.13	$17.58
Societal burden^c^ (in million USD)					
PCV10 serotypes	$0.31	$0.33	$0.23	$0.21	$0.81
PCV13 serotypes	$0.53	$3.32	$1.95	$2.23	$10.35
PCV15 serotypes	$0.58	$3.32	$2.40	$2.23	$10.76
PCV20 serotypes	$0.81	$4.66	$2.88	$2.31	$12.63
Higher assumed proportion of *S. pneumoniae*					
Clinical burden^a^					
PCV10 serotypes	25,118	12,555	6,585	11,358	19,029
PCV13 serotypes	42,307	125,555	55,345	117,922	243,565
PCV15 serotypes	46,237	125,555	68,044	117,922	253,291
PCV20 serotypes	64,704	176,120	81,841	122,431	297,268
Economic burden^b^ (in million USD)					
PCV10 serotypes	$3.00	$12.47	$0.36	$2.65	$2.84
PCV13 serotypes	$5.04	$124.67	$2.99	$27.53	$36.41
PCV15 serotypes	$5.51	$124.67	$3.67	$27.53	$37.86
PCV20 serotypes	$7.72	$174.87	$4.41	$28.58	$44.44
Societal burden^c^ (in million USD)					
PCV10 serotypes	$0.80	$0.49	$0.61	$0.56	$2.09
PCV13 serotypes	$1.35	$0.82	$5.11	$5.77	$26.69
PCV15 serotypes	$1.47	$0.90	$6.27	$5.77	$27.76
PCV20 serotypes	$2.06	$1.26	$7.56	$5.99	$32.58

NIP, national immunization program; PCV, pneumococcal conjugate vaccine, USD, United States Dollar.

a Clinical burden inclusive of total cases of each pneumococcal disease state, including IPD, inpatient pneumonia, outpatient pneumonia, and OM.

b Economic burden inclusive of direct medical costs due to each pneumococcal disease state, including IPD, inpatient pneumonia, outpatient pneumonia, and OM.

c Societal burden inclusive of indirect costs due productivity loss resulting from each pneumococcal disease state, including IPD, inpatient pneumonia, outpatient pneumonia, and OM.

## 4 Discussion

This study demonstrates that there remains substantial clinical, economic, and societal burden due to pneumococcal disease children aged <5 years old in Argentina, Mexico, Chile, Colombia, and Brazil. In total, across all five countries, PCV20 serotypes were estimated to cause over 510,000 pneumococcal disease cases and 2,700 deaths, resulting in economic burden of over $182 million and societal burden of over $34 million. While IPD can cause serious illness, non-invasive disease remains highly prevalent and was estimated to account for the majority of pneumococcal disease burden in each country. These results demonstrate the importance of maintaining pneumococcal vaccination for children and expanding access to higher-valent vaccines such as PCV20 where feasible.

PCV13-type disease resulted in substantial burden in each of the five countries included in this analysis. In the PCV10 NIP countries, the model estimated most of the PCV20-type burden was due to the PCV13-10 serotypes. PCV10 has been successful in reducing the incidence of vaccine-type serotypes, but many countries which used PCV10 report high incidence of disease caused by serotypes 6A and 19A (45–50). However, in all five countries included in this analysis, PCV15-13 serotypes caused minimal burden beyond PCV13, while PCV20-15 serotypes were estimated to cause substantially more disease cases and deaths. Protection against PCV13 serotypes, while also expanding valency to emerging serotypes included only in PCV20, could improve public health and alleviate economic and societal burden in these countries.

The analysis is driven by country-specific serotype distribution, which is influenced by each country's historic NIP recommendations and vaccine uptake. In Argentina, PCV13 has been recommended since 2012 and has had a great impact on disease incidence, but disease due to serotypes 1, 3, 14, and 19A still persists (4, 13). In recognition of the potential public health benefits of PCV20, Argentina introduced PCV20 into the pediatric NIP in 2025 (51). However, vaccination rates for most major childhood vaccines in Argentina have been steadily declining since 2014, from close to or above 90% to 71% in 2023 (52). Ensuring high uptake and adherence to the full PCV20 dosing schedule is essential for sustained protection against pneumococcal disease.

Mexico introduced PCV7 into its NIP in 2006, followed by PCV13 in 2010 (27). While reductions in IPD mortality and overall IPD incidence were observed post-PCV13 implementation, our analysis indicates that disease burden from PCV13 serotypes persists (5). This may be attributed to suboptimal vaccine coverage among children (below the government's target of 90%) (54). Furthermore, data from 2017 demonstrated that Mexican children may not be completing their infant vaccination schedule on time, nor were they consistently receiving a booster dose during toddlerhood (9, 53, 54)]. These factors may contribute to decreased levels of protection against PCV13-type disease. As recommended in Argentina, PCV20 vaccination could continue to protect against PCV13 serotypes and expand protection due to its broader serotype coverage.

Chile adopted PCV10 in its NIP in 2011 then switched to PCV13 in 2017, reaching 93% vaccine coverage by 2022 (52, 55). Despite this, our analysis estimated about 38,000 cases per year were caused by PCV13 serotypes, and PCV20-unique serotypes were estimated to cause nearly 50% more annual cases than PCV13. Since the serotype data used in the analysis is only representative of two years of PCV13 use, these estimates may not fully reflect current epidemiology. However, introducing a higher-valent vaccine in Chile could substantially lower the clinical, economic, and societal impact of pneumococcal disease.

Brazil introduced PCV10 to its pediatric NIP in 2010, and while PCV13 was also licensed in 2019, it was only recommended for people aged >5 years with high-risk conditions (34). The analysis estimated a minimal proportion of disease burden was due to the PCV10 serotypes in Brazil, likely due to the long-term PCV10 NIP and relatively high vaccination coverage (estimated at 81% in 2022) (52). Most of the remaining burden of pneumococcal disease in Brazil is due to serotypes in higher-valent vaccines, which was recognized by the Brazilian Ministry of Health following their decision to implement PCV20 into their pediatric NIP from January 2026 (56).

Colombia implemented PCV10 into its pediatric NIP in 2012, though made the decision to switch to PCV13 in 2022 (57). However, because the burden estimates were based on serotype coverage prior to 2022, the impact of PCV13 is not reflected in the results. PCV13 serotypes were estimated to cause of 81,000 cases in Colombia, therefore PCV13 implementation may result in significant reductions in disease burden. Future decisions regarding the implementation of higher-valent PCVs in Colombia should be informed by epidemiology data which is representative of the updated PCV13 NIP.

This study has several limitations, primarily due to the availability and quality of epidemiology data used in the model. While SIREVA provides robust information on key bacterial pathogens causing disease, antimicrobial resistance, and circulating pneumococcal serotypes, the surveillance is passive and may not fully capture the true epidemiologic situation in each country. A recent analysis of SIREVA data from six Latin American countries compared the number of IPD isolates in children <5 years old estimated that the number of isolates reported in SIREVA was consistently lower than the actual number of cases (22). While the data used in this analysis suggest that serotypes included in higher-valent vaccines constitute a large proportion of the estimated vaccine-type disease burden, serotype patterns may have shifted and transmission dynamics may have changed following the COVID-19 pandemic (13). Active IPD surveillance is vital to ensure policy decisions regarding pediatric pneumococcal vaccination is data-driven and targeting the most prevalent disease-causing serotypes.

Additionally, the sources used to derive incidence estimates have heterogeneity related to population size, study design, and time, so they may not be nationally representative of a country's epidemiology. In addition to this, due to limited data regarding the serotype distribution of non-invasive disease, the serotype distribution of pneumococcal OM and pneumonia was assumed to be the same as that of IPD. Invasive disease cases are more likely to be serotyped than non-invasive disease cases due to surveillance reporting requirements and there is limited evidence to inform an assumption that the serotype distribution of invasive and non-invasive disease is different. Despite limitations with the input data, probabilistic sensitivity analysis (PSA) was not conducted for this analysis because the model was designed as a deterministic nature and many key epidemiological and cost inputs were sourced as a point of estimates, and parameter distributions were not available. Therefore, it was not feasible to conduct PSAs. However, scenario analyses demonstrated that varying the proportion of OM and pneumonia attributable to *S. pneumoniae* produced directionally consistent changes in clinical outcomes, direct medical costs, and indirect costs. While absolute burden estimates are sensitive to this parameter, the overall pattern of results and relative comparisons across vaccines remained stable.

Although real-world evidence has demonstrated that both PCV10 and PCV13 have resulted in pneumococcal disease reductions, there are currently no published studies that report the effectiveness of higher-valent PCVs in children (58). This analysis does not present estimates of prevented cases, but rather the number of cases that could be caused by PCV15 and PCV20 serotypes. While PCV15 and PCV20 may further reduce pneumococcal disease, there are numerous considerations beyond disease burden related to budget, access, and uptake that should be accounted for when switching to a higher-valent vaccine. The landscape is likely to be rapidly evolving, with varied vaccination policies in the region currently at different maturation points. Therefore, extrapolating the results of this analysis to other countries or the entire continent might not be feasible.

## 5 Conclusion

This study highlights that PCV20 serotypes are responsible for a considerable clinical, economic, and societal burden of pneumococcal disease in children <5 years old across Argentina, Chile, Mexico, Colombia, and Brazil. Although IPD is associated with more severe outcomes, non-invasive diseases such as pneumonia and OM constituted the majority of disease burden and can impact thousands of families each year. Implementation of higher-valent PCVs that elicit sustained protection against PCV13 serotypes but also offer protection against emerging serotypes may result in improved public health outcomes while also alleviating a substantial economic and societal toll.

## Data Availability

The original contributions presented in the study are included in the article/Supplementary Material, further inquiries can be directed to the corresponding author/s.
