# Supplementary material for: Estimated pneumococcal disease burden in children due to serotypes covered by different pneumococcal conjugate vaccines in five Latin American countries
Source: Front Pediatr. 2026 May 21;14:1768403. doi: 10.3389/fped.2026.1768403 (PMC13233521; doi:10.3389/fped.2026.1768403)
Supplement: Supplementary file 1 [file Datasheet1.pdf]

Supplementary materials

1 Model calculations

If the incidence rate was based on invasive pneumococcal disease or pneumococcal pneumonia or OM, the total number of pneumococcal disease cases is calculated using the below formula:

$$\textit{Number of cases} = \textit{Population size} \times \textit{Serotype coverage} \times \textit{Disease incidence rate}$$

Where the population size is total number of children in the specified age group, serotype coverage is the proportion of disease caused by serotypes included in the vaccine (expressed as a decimal; e.g., 20% as 0.2), and the disease incidence rate is the number of cases per 100,000 (expressed as a decimal; e.g., 10 per 100,000 as 0.0001)

If the incidence rate for non-invasive pneumococcal disease cases is based on all-cause, the total number of pneumococcal disease cases is calculated using the below formula:

$$\textit{Number of cases} = \textit{Population size} \times \textit{Serotype coverage} \times \textit{Disease incidence rate based on all-cause} \times \textit{the proportion of pneumococcal disease of the all-cause}$$

Where the proportion of pneumococcal pneumonia and OM of all-cause pneumonia and OM was assumed to be 29.1% (1).

The number of disease-related deaths are calculated by multiplying the number of cases by the disease-specific case fatality rate (CFR), expressed as a decimal:

$$\textit{Number of deaths} = \textit{Number of cases} \times \textit{CFR}$$

To determine the economic burden, the model then multiplies the number of disease cases by the direct medical costs for treating a single case, using the following formula:

$$\textit{Total direct costs} = \textit{Number of cases} \times \textit{Direct medical cost per case}$$

For clarity, a hypothetical calculation of the clinical and economic burden in a population of 2,000,000 children, in which serotype coverage is 20% (0.2), disease incidence rate is 10 per 100,000 (0.0001), 5% of cases result in fatality, and the cost of treating a case is $1,000 would be as follows:

$$\textit{Number of cases} = 2,000,000 \times 0.2 \times 0.0001 = 40$$

$$\textit{Number of deaths} = 40 \times 0.05 = 2$$

$$\textit{Total direct costs} = 40 \times \$1,000 = \$40,000$$

2 Indirect non-medical cost calculations

For each hospitalized condition, we first calculated the number of work hours a caregiver could miss while their child is in hospital, assuming caregivers missed work for the entirety of the child's hospital stay, using average length of stay (ALOS) data. IPD and inpatient pneumonia ALOS were sourced from country-specific published data (Supplementary Table 1). If local data were not available, we assumed the IPD LOS was 12.1 days based on the

ALOS for pneumococcal sepsis and meningitis from the 2007 Sabin Vaccine Institute Report and inpatient pneumonia ALOS was 10 days based on a meta-analysis of pediatric pneumococcal pneumonia in the region by Bardach et al. (2017)(2, 3). Following a methodology by Wilson et al. (2020), an additional loss of 5 workdays was added for time off required to care for the child after hospitalization had ended (4). The number of work hours missed was calculated using the below formula:

Work hours missed = (Average length of stay (ALOS) + 5 days) x Average daily working hours

The average daily working hours in each country was informed by data from the International Labour Organization Department of Statistics (ILOSTAT) (5). For example, if the IPD ALOS was 9 days and the caregiver spent 7 hours a day at work, the calculation for work hours missed would be as follows:

Work hours missed = (9 days + 5 days) x 7 working hours/day = 98

To estimate total hours of lost productivity, we considered there may be a proportion of caregivers who do not work using data from the World Bank on the percentage of population in the workforce (6). To account for this in the model, we used the following formula:

Total hours of lost productivity = Work hours missed x Percentage of population in the workforce

The below calculation estimates the total hours of lost productivity using the previous example if 60% of the population is in the workforce:

Total hours of lost productivity = 98 work hours missed x 0.6 = 59

The total hours of lost productivity per disease case are reported in Supplementary Table 1.

For all countries, we assumed caregivers lost 5 workdays for outpatient pneumonia and 1 workday for AOM, based on an analysis conducted in Brazil by Sartori et al. (2012) (7). To calculate indirect costs per case, we assumed the cost of a missed working hour to be equivalent to the average hourly wage for each country, reported by ILOSTAT and converted to 2023 USD (8). The indirect cost per each case of pneumococcal disease was calculated using the following formula:

Indirect cost per case = Total hours of lost productivity x Hourly wage

Using the previous example, if the average hourly wage was $5 USD, the indirect cost per case would be calculated as follows:

Indirect cost per case = 59 hours x $5/hour = $295

To estimate the societal burden associated with each disease state, the indirect cost per disease episode was then multiplied by the respective number of cases per disease state. Societal burden estimates are presented in Table 1 of the main text.

Supplemental Table 1. Estimates of productivity loss per case.

	Argentina	Brazil	Chile	Colombia	Mexico
**IPD**[a]					
ALOS (days)	9.1(9)	10.0 (10)	7.0 (11)	13.6 (12)	14.0 (13)
Caregivers absent from work (%)[a]	60.4	61.9	57.1	66.7	58.8
Hours of productivity lost[b]	59.8	65.2	47.4	95.8	86.7
**Inpatient pneumonia**[a]					
ALOS (days)	9.5 (9)	5.0(10)	5.0(11)	12.0(14)	10.0 (2)
Caregivers absent from work (%)	60.4	61.9	57.1	66.7	58.8
Hours of productivity lost	61.5	43.5	39.5	25.8	68.4
**Outpatient pneumonia**[b]					
Caregivers absent from work (%)	60.4	61.9	57.1	66.7	58.8
Hours of productivity lost	21.2	21.7	19.8	25.8	22.8
**OM**[c]					
Caregivers absent from work (%)	60.4	61.9	57.1	66.7	58.8
Hours of productivity lost	4.2	4.3	4	5.2	4.6

ALoS, average length of stay; OM, otitis media; IPD, invasive pneumococcal disease.

[a]Assumed 100% of children are hospitalized.

[b]Assumed 0% of children are hospitalized, caregivers miss 5 days of work.

[c]Assumed 0% of children are hospitalized, caregivers miss 1 day of work.

Supplemental Table 2. Serotype-specific estimates of disease coverage by PCV.

	PCV13 NIP countries			PCV10 NIP countries	
**Serotype**	**Argentina**	**Mexico**	**Chile**	**Brazil**	**Colombia**
4	0.0%	0.0%	0.0%	1.1%	0.7%
6B	0.2%	1.2%	0.0%	0.0%	1.3%
9V	2.2%	0.0%	0.7%	1.1%	0.0%
14	4.9%	0.0%	1.4%	0.6%	3.4%
18C	0.7%	0.0%	0.0%	0.0%	0.7%
19F	1.2%	0.0%	0.7%	0.6%	0.0%
23F	0.5%	2.4%	0.7%	0.0%	0.7%
1	4.9%	0.0%	0.0%	0.0%	0.0%
5	1.2%	0.0%	0.0%	0.0%	0.0%
7F	2.2%	0.0%	0.7%	1.1%	0.0%
3	7.4%	4.9%	12.8%	7.4%	10.1%
6A	0.0%	0.0%	0.7%	1.1%	2.7%
19A	4.9%	28.0%	17.6%	44.6%	51.0%
22F	2.9%	0.0%	3.4%	2.3%	0.0%
33F	0.0%	0.0%	4.7%	0.0%	0.0%
8	0.0%	0.0%	0.7%	0.6%	0.0%
10A	0.0%	3.7%	3.4%	2.9%	0.0%
11A	1.5%	2.4%	0.7%	0.6%	0.7%
12F	9.6%	0.0%	2.0%	4.0%	0.0%
15B	2.2%	8.5%	2.0%	2.3%	2.0%

Supplemental Table 3. Estimated annual economic burden due to pneumococcal disease cases, by disease state, attributable to the serotypes included in PCV10, PCV13, PCV15, and PCV20 (in million USD).

	PCV10	PCV13	PCV15	PCV20
**Argentina**				
Total	$2.13	$3.59	$3.93	$5.50
IPD	$0.22	$0.37	$0.41	$0.57
Inpatient pneumonia	$1.37	$2.31	$2.53	$3.54
Outpatient pneumonia	$0.04	$0.06	$0.07	$0.09
OM	$0.50	$0.85	$0.93	$1.29
**Mexico**				
Total	$8.73	$87.28	$87.28	$122.44
IPD	$0.42	$4.24	$4.24	$5.95
Inpatient pneumonia	$3.63	$36.31	$36.31	$50.94
Outpatient pneumonia	$2.30	$22.97	$22.97	$32.22
OM	$2.38	$23.76	$23.76	$33.32
**Chile**				
Total	$0.25	$2.07	$2.55	$3.06
IPD	$0.01	$0.04	$0.05	$0.06
Inpatient pneumonia	$0.03	$0.29	$0.35	$0.42
Outpatient pneumonia	$0.14	$1.19	$1.46	$1.76
OM	$0.07	$0.55	$0.68	$0.82
**Colombia**				
Total	$1.89	$19.60	$19.60	$20.35
IPD	$0.19	$2.00	$2.00	$2.08
Inpatient pneumonia	$1.45	$15.04	$15.04	$15.61
Outpatient pneumonia	$0.19	$1.98	$1.98	$2.06
OM	$0.06	$0.58	$0.58	$0.60
**Brazil**				
Total	$1.98	$25.33	$26.34	$30.91
IPD	$0.08	$0.96	$1.00	$1.18
Inpatient pneumonia	$1.35	$17.23	$17.92	$21.03
Outpatient pneumonia	$0.46	$5.89	$6.13	$7.19
OM	$0.10	$1.24	$1.29	$1.51

OM, otitis media; IPD, invasive pneumococcal disease; PNE, pneumonia

Supplemental Table 4. Estimated annual societal burden due to pneumococcal disease cases, by disease state, attributable to the serotypes included in PCV10, PCV13, PCV15, and PCV20 (in million USD).

	PCV10	PCV13	PCV15	PCV20
**Argentina**				
Total	$0.56	$0.94	$1.02	$1.43
IPD	$0.02	$0.03	$0.03	$0.05
Inpatient pneumonia	$0.02	$0.04	$0.04	$0.06
Outpatient pneumonia	$0.33	$0.55	$0.61	$0.85
OM	$0.56	$0.94	$1.02	$1.43
**Mexico**				
Total	$0.59	$5.91	$5.91	$8.29
IPD	$0.02	$0.16	$0.16	$0.22
Inpatient pneumonia	$0.15	$1.54	$1.54	$2.16
Outpatient pneumonia	$0.41	$4.11	$4.11	$5.76
OM	$0.01	$0.11	$0.11	$0.15
**Chile**				
Total	$0.42	$3.53	$4.34	$5.22
IPD	$0.00	$0.02	$0.02	$0.03
Inpatient pneumonia	$0.01	$0.12	$0.15	$0.18
Outpatient pneumonia	$0.33	$2.79	$3.43	$4.12
OM	$0.07	$0.60	$0.73	$0.88
**Colombia**				
Total	$0.39	$4.00	$4.00	$4.15
IPD	$0.01	$0.06	$0.06	$0.06
Inpatient pneumonia	$0.17	$1.74	$1.74	$1.81
Outpatient pneumonia	$0.19	$2.00	$2.00	$2.08
OM	$0.02	$0.20	$0.20	$0.21
**Brazil**				
Total	$1.44	$18.46	$19.20	$22.53
IPD	$0.03	$0.37	$0.38	$0.45
Inpatient pneumonia	$0.91	$11.65	$12.12	$14.22
Outpatient pneumonia	$0.48	$6.18	$6.43	$7.54
OM	$0.02	$0.26	$0.27	$0.32

OM, otitis media; IPD, invasive pneumococcal disease.

3 References

1. Ngo CC, Massa HM, Thornton RB, Cripps AW. Predominant Bacteria Detected from the Middle Ear Fluid of Children Experiencing Otitis Media: A Systematic Review. PLoS One. 2016;11(3):e0150949.
2. Bardach AE, Rey-Ares L, Calderon Cahua M, Ciapponi A, Cafferata ML, Cormick G, et al. Burden of culture-confirmed pediatric pneumococcal pneumonia in Latin America and the Caribbean: A systematic review and meta-analysis. Value in Health Regional Issues. 2017;14:41-52.
3. Constenla D, Gómez E, De la Hoz F, O'Loughlin R, Sinha A, Valencia J, et al. The burden of pneumococcal disease and cost-effectiveness of a pneumococcal vaccine in Latin America and the Caribbean. Sabin Vaccine Institute. 2007;1:129.
4. Wilson MR, Wasserman MD, Breton MC, Peloquin F, Earnshaw SR, McDade C, et al. Health and economic impact of routine pediatric pneumococcal immunization programs in Canada: A retrospective analysis. Infectious Diseases and Therapy. 2020;9(2):341-53.
5. International Labor Organisation Statistics. Statistics on Working Time. 2020.
6. The World Bank. Labor force participation rate, total (% of total population ages 15+)(national estimate). 2022.
7. Sartori AL, Minamisava R, Bierrenbach AL, Toscano CM, Afonso ET, Morais-Neto OL, et al. Reduction in all-cause otitis media-related outpatient visits in children after PCV10 introduction in Brazil. PLOS ONE. 2017;12(6):e0179222.
8. International Labor Organisation Statistics. SDG Indicator 8.5.1 – Average hourly earnings of employees by sex (Local currency) – Annual. 2020.
9. López EL, Glatstein E, Ezcurra GC, Iacono M, Teplitz E, Garnero AV, et al. Rapid decrease in rates of hospitalization resulting from invasive pneumococcal disease and community-acquired pneumonia in children aged <60 months after 13-valent pneumococcal conjugate vaccine introduction in Argentina. Journal of the Pediatric Infectious Disease Society. 2018;7(1):30-5.
10. Sartori AM, de Soárez PC, Novaes HM. Cost-effectiveness of introducing the 10-valent pneumococcal conjugate vaccine into the universal immunisation of infants in Brazil. Journal of Epidemiolpgy and Community Health. 2012;66(3):210-7.
11. Alvarado S, Cavada G, Villena R, Wilhelm J, Budnik I, Lara C, et al. Impact of the 10-valent pneumococcal conjugate vaccine on the southern area of Santiago (Chile), 2009-2015. Revista Panamericana de Salud Publica. 2018;42:e155.
12. Camacho Moreno G, Imbachi LF, Leal AL, Moreno VM, Patiño JA, Gutiérrez IF, et al. Emergence of Streptococcus pneumoniae serotype 19A (Spn19A) in the pediatric population in Bogotá, Colombia as the main cause of invasive pneumococcal disease after the introduction of PCV10. Human Vaccines & Immunotherapeutics. 2020;16(9):2300-6.
13. Chacon-Cruz E, Rivas-Landeros RM, Volker-Soberanes ML. Early trends in invasive pneumococcal disease in children following the introduction of 13-valent pneumococcal conjugate vaccine: results from eight years of active surveillance in a Mexican hospital. Ther Adv Vaccines. 2014;2(6):155-8.
14. Gutiérrez-Tobar IF, Londoño-Ruiz JP, Mariño-Drews C, Beltrán-Higuera S, Camacho-Moreno G, Leal-Castro AL, et al. Epidemiological characteristics and serotype distribution of culture-confirmed pediatric pneumococcal pneumonia before and after PCV 10 introduction, a multicenter study in Bogota, Colombia, 2008–2019. Vaccine. 2022;40(20):2875-83.
